# Effect of Eco-Friendly Cellulose Nanocrystals on Physical Properties of Cement Mortars

**DOI:** 10.3390/polym11122088

**Published:** 2019-12-13

**Authors:** Danuta Barnat-Hunek, Małgorzata Grzegorczyk-Frańczak, Monika Szymańska-Chargot, Grzegorz Łagód

**Affiliations:** 1 Faculty of Civil Engineering and Architecture, Lublin University of Technology, Nadbystrzycka 40, 20-618 Lublin, Poland; m.grzegorczyk@pollub.pl; 2 Institute of Agrophysics PAS, Doswiadczalna 4, 20-290 Lublin, Poland; m.szymanska@ipan.lublin.pl; 3 Faculty of Environmental Engineering, Lublin University of Technology, Nadbystrzycka 40B, 20-618 Lublin, Poland; g.lagod@pollub.pl

**Keywords:** nanocellulose, nanocrystals, cement mortar, microstructure, physical properties, moisture related properties, frost resistance

## Abstract

Nanocellulose, being a material with nanodimensions, is characterized by high tensile strength, high modulus of elasticity, low thermal expansion, and relatively low density, as well as exhibiting very good electrical conductivity properties. The paper presents the results of research on cement mortars with the addition of nanocrystals cellulose, applied in three different amounts (0.5%, 1.0%, and 1.5%) by weight of cement, including: physical and mechanical properties, frost resistance and resistance against the detrimental effect of salt, and microstructure examination (SEM). Along with an increase in amount of admixture, the weight loss following frost resistance and salt crystallization tests is reduced. Studies have shown that the addition of nanocrystalline cellulose improves the compressive and flexural strength by 27.6% and 10.9%, respectively. After 50 freezing and thawing (F–T) cycles for the mortars with 1.5% nanocellulose admixture, an improvement in frost resistance by 98% was observed. In turn, the sulfate crystallization tests indicated a 35-fold decrease in weight loss following 1.5% nanopolymer addition to the mortar.

## 1. Introduction

At present, nanotechnology is one of the most dynamically developing interdisciplinary fields of science and technology. It combines issues related to physics, chemistry, biology, material engineering, and bioengineering. It pertains to the materials in molecular scale up to 100 nm, at least in one dimension, which are characterized by different properties from their macroscopic chemical counterparts due to their quantum effects [1].

Nanotechnology enables the design, modification, and manufacture of nanometric materials. Nanotechnology employs the bottom-up (self-organization of particles) and top-down (reduction to nanoparticles) methods, surface modification, and structure modification using admixtures of nanomodificators [2]. Nanomaterial technology has been adopted in civil engineering, especially in construction chemicals for modifying building materials. Introduction of nanosilver admixture to a mortar mix for grouting ceramic tiles confers the bactericidal effect of nanosilver to the grout [3]. Introduction of dispersed TiO_2_ nanomolecules to concrete enabled the obtaining of a material with a self-cleaning surface [4]. The studies by Praveenkumar et al. [5] on cement concrete with the addition of TiO_2_ nanomolecules and 10% rice husk enabled the obtaining of a material with greater compressive and flexural strength, as well as enhanced resistance to the effect of chlorides and acids. The addition of nanosilica [6] accelerates the cement hydration process and improves the compressive strength of concrete at the initial stages of maturation. Moreover, the samples with nanosilica exhibited higher resistance to corrosion in the presence of sodium sulfate. Nanosilica added to cement materials acts as nanofiller, which greatly improves the interfacial transition zone [7].

The cement mortars modified with nanomagnesium calcite exhibit a greater resistance against the effect of magnesium sulfate [8]. This is because the infiltration of sulfate to the mortar is retarded, as a result of reduced pore volume that occurs due to the nanomaterial application. The research conducted by Stankiewicz and Lelusz [9] proved the beneficial influence of carbon nanotube addition to cement mortar in the amount of 0.06% by weight, which resulted in the enhancement of the flexural and compressive strength by 25% and 36%, respectively, in relation to the reference mortars. The mortars with nanoaluminum addition exhibit fire-resistant properties [10]. Szymanski and Sadowski [11] showed that the addition of alumina nanopowder (Al_2_O_3_) to cement mortar improves substrate adhesion. In the studies by Irshidat et al. [10] the mortars were characterized by 15% greater compressive strength in comparison to the reference mortars subjected to thermal treatment at 600 °C. Kropyvnytska et al. [12] indicated a positive influence of nano-liquids on the operational properties of brick buildings.

In recent years, scientists from all around the world have emphasized modification of materials using eco-friendly additions and admixtures, so-called biopolymers. They have always been present in the environment and constitute an ecological alternative for synthetic polymer admixtures. Examples of natural polymers include keratin, collagen, chitin and chitosan, starch, lignin, or cellulose. Cellulose is the most abundant biopolymer on Earth; it can be harvested from wood, cotton, or plant biomass, through the synthesis of algae and bacteria as well as tunicates. It is produced through photosynthesis and constitutes a basic plant ingredient. It is a linear biopolymer and is composed from glucopyranose residues, in which mers are linked with 1,4-β-glycosidic bonds. Each of the heterocyclic rings occurring in cellulose contains: a primary hydroxyl group (–CH_2_OH) and two secondary hydroxyl groups (–OH) [13]. When cellulose chains are linked into bundles forming domains, it is possible to extract them as nanomolecules. Then, they exhibit special properties resulting from their size (nanoscale), fiber morphology, and large specific surface. Cellulose nanomaterials can be divided depending on the source and obtaining method, and chemical properties of surface and can be classified into five categories: cellulose nanocrystals (CCNC), cellulose nanofibrils (CNF), tunicate cellulose nanocrystals (t-CNC), algae cellulose (AC), and bacterial cellulose (BC) [14,15,16]. The plant-derived nanocellulose is used to manufacture adhesive additives, drilling fluids, cement-based materials, food coating, transparent and flexible electronics, structures enhancing the performance of catalysis, and biomedical materials [17]. Numerous studies were also devoted to the application of nanocellulose in the water and wastewater treatment process [18,19].

Most frequently, the nanocrystalline cellulose is obtained from wood pulp and cotton [20]; however, starting materials such as rice husk, pineapple, coconut shells, banana, or carrot—which was used in this study to derive CCNC—are becoming increasingly popular. The selection of the material for cellulose acquisition and the production process influence the dimensions, surface activity and the properties of the obtained material [21,22]. CCNC is obtained through acidic hydrolysis of cellulose fibers. Sulfuric acid is the most commonly acid employed for the CCNC isolation. The most recent CCNC isolation methods involve oxidation and hydrolysis with various acids, including hydrochloric, hydrobromic, citric, phosphoric, oxalic, and maleic [20]. The acid selection influences the properties of the obtained nanomaterial. The CCNC isolated by means of sulfuric or phosphoric acids have a negative surface charge, which results in electrostatic stabilization of suspensions of these nanocrystals [23].

This paper involved research on cement mortars prepared using nanocrystalline cellulose admixtures. It is a highly porous (≥99%) nanomaterial, which is easily obtained by releasing frozen water from aqueous suspensions through lyophilization of nanofibril cellulose (NFC). Nanocrystalline cellulose is characterized by high tensile strength, high modulus of elasticity, low thermal expansion, and relatively low density, as well as exhibiting very good electrical conductivity properties [24]. In recent years it has been investigated as a prospective additive in cement materials. It was indicated that CCNC, as an additive to cement materials, improves the mechanical properties [25,26], increases the hydration degree [26], as well as enhances the microstructure of cement composites [27]. The presented article aimed to examine and assess the properties exhibited by cement mortars, both with and without the CCNC admixtures. To the best of the authors’ knowledge, there are no studies on the durability of cement mortars with nanocellulose addition, especially their frost resistance and resistance to sulfides. The assessment was carried out using mortars with varying content of CCNC (0.5%, 1%, and 1.5% by weight). The investigation involved the physical and mechanical properties of mortars; their microstructure was analyzed as well.

## 2. Materials and Methods

### 2.1. Mortars Composition

The compositions of the mortars as well as their abbreviated names are presented in Table 1. The mortars were designated in the following way: SM—standard mortars, no admixtures, CM—cement mortars with the addition of carrot cellulose nanocrystals (CCNC), 0.5, 1, and 1.5, the percentage of admixture by weight of cement. The assumed water to cement ratio (*w*/*c*) equaled 0.45.

Quartz sand (0–2 mm) is characterized by specific gravity of 2650 kg/m^3^; water absorption amounts to 1.2%, while the moisture content equals 0.16% [28]. Table 2 presents the CEM I 32.5R Portland cement parameters. This cement was designed in accordance with the Polish standards, namely PN-EN 197-1:2012 [29] and PN-B-19707:2013-10 [30].

### 2.2. Cellulose Nanocrystals

The carrot cellulose was obtained by subjecting carrot pomace to thermochemical processes to remove the non-cellulose polymers [32]. The cellulose nanocrystals were prepared through high-intensity ultrasonication, in line with the technique described by Szymańska-Chargot et al. [33,34].

The ultrasonication system comprised a Sonics Vibracell ultrasonic homogenizer (VCX-130FSJ; Sonics & Materials, Inc., Newtown, CT, USA), temperature probe; additionally, an ice bath was used and to avoid heating the samples. The operation amplitude of ultrasonic homogenizer was maintained at 90% of the nominal amplitude. Each time, 250 g of cellulose dispersion in concentration of 0.1 wt % was introduced to ultrasound treatment of 60 min. Studies proved that this treatment enables the preparation of cellulose nanocrystals from carrots in the form of whiskers (CCNC) [33]. Afterwards, the obtained CCNC was concentrated to approx. 5 wt % by means of vacuum filtration. The CCNC was characterized by molecular structure (FT-IR), crystallinity degree evaluated (XRD) method, and AFM images.

A Nicolet 6700 FT-IR spectrometer (Thermo Scientific, Waltham, MA, USA) with the Smart iTR ATR sampling accessory was used to collect the FT-IR spectra of CCNC. CCNC was placed on attenuated total reflectance (ATR) crystal as a lyophilized powder. The spectra were recorded in the range of 4000–650 cm^−1^ in five replications and 200 scans were taken per spectrum. All spectra were measured at a spectral resolution of 4 cm^−1^. The presented FT-IR spectrum is the average value of the respective single spectra.

The degree of CCNC crystallinity was determined with the X-ray diffraction method. The X-ray diffractometer Empyrean (PANalytical, Almelo, The Netherlands) was used. The investigated samples were scanned with Cu Kα radiation (λ = 0.15418 nm). The parameters of the working lamp were as follows: *U* = 40 kV, *I* = 25 mA. The intensity of reflections was measured over the angular 5–90° 2θ with step intervals of 0.05°. The duration of the reflection count was 10 s. Based on the registered measurements, a mathematical model describing the relationship between intensity and 2θ was developed. The degree of crystallinity was calculated according to the Segal method [35,36]:

CI = (*I*_max_ − *I*_min_) × 100%/*I*_max_ (1)

where CI—degree of crystallinity; *I*_max_—the intensity value for the crystalline cellulose (2θ = 22.5°); and *I*_min_—the intensity value for the amorphous cellulose (2θ = 18°).

### 2.3. Methods

The preparation of samples was carried out in accordance with PN-EN 196-1-2016 standard [37]. All components were mixed for 2 min in a mortar mixer. Then, water and CCNC were added and mixed for 2 min. The molds were filled to the half of their capacity and compacted for 1 min by means of a vibrating table Another layer of mixtures was laid and the samples were compacted again. The 40 mm × 40 mm × 160 mm samples were placed in molds and left for 24 h under the conditions stated in the afore-mentioned standard. Afterwards, the samples were demolded and placed for 21 days in a climatic chamber characterized by the temperature of 23.5 °C and relative humidity of 73.5%. After this period, the samples were dried to constant mass and subjected to investigations.

**Figure 1:**
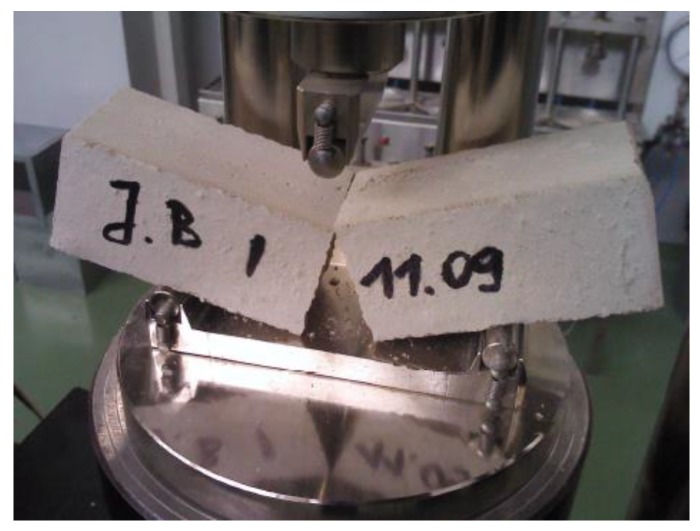
Flexural strength in bending test of CM1.

**Figure 2:**
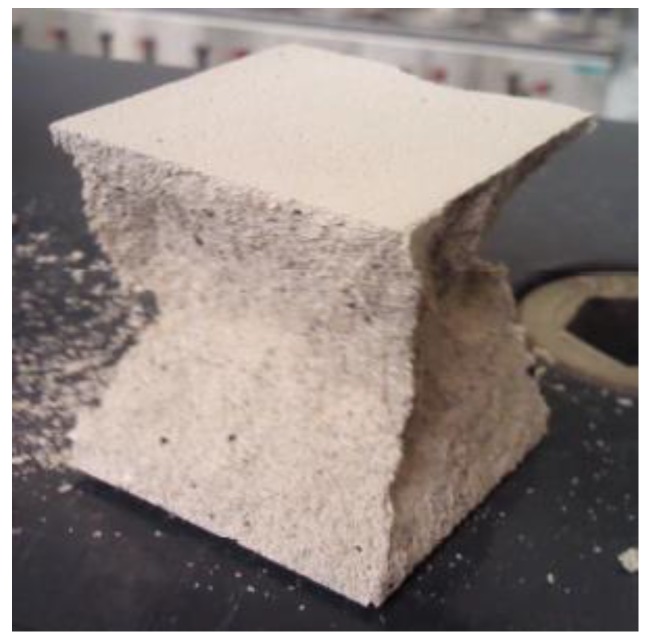
CM1 sample destruction following the compressive strength test.

Specific density and bulk density, as well as total porosity were determined in line with PN-EN 1936:2010 [38] on the previously dried samples. The flexural strength characterizing the rectangular prisms of cement mortars with the dimensions of 40 mm × 40 mm × 160 mm (Figure 1) was measured in accordance with BS EN 1015-11:2002 standard [39]. The samples were tested following 28 days of curing, being loaded with centrally applied force (3-point bending). The compressive strength was determined in accordance with [39] standard, using the samples which underwent the flexural strength test (Figure 2). Load was applied centrally at 0.05 MPa/s (Advantest 9, Controls S.p., Milan (MI), Italy).

**Figure 3:**
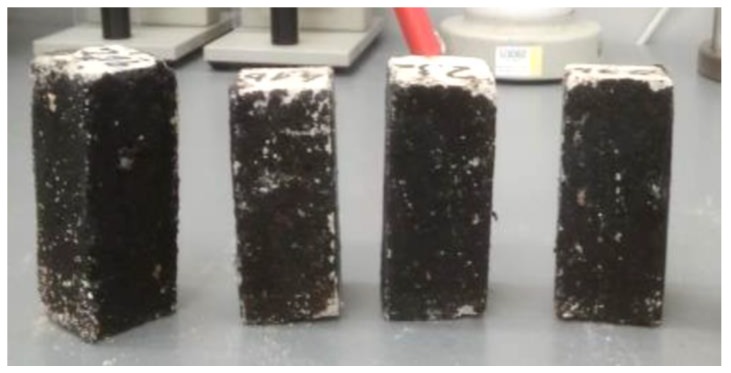
CM1.5 mortar samples used to determine the water absorption coefficient *C*_m_.

**Figure 4:**
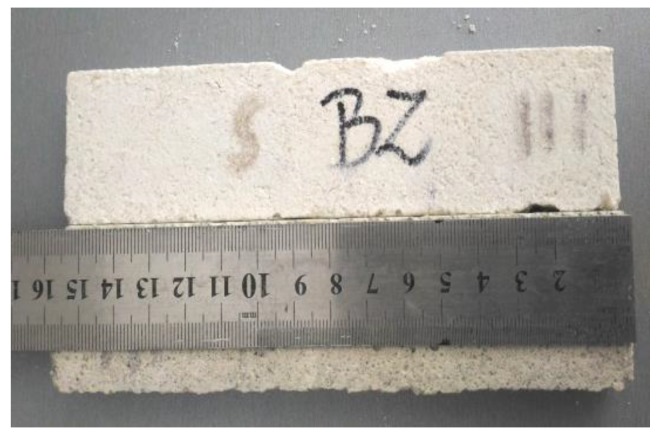
Determination of the water absorption coefficient *C*_m_ of the CM1.5 mortar.

The BS EN 13755:2008 standard [40] was used for testing the absorptivity of the investigated cement mortars. The influence of moisture on the considered hydrophobized mortars was examined in 1-, 7-, and 14-day intervals to indicate the effect of moisture. The cup method described in PN-EN ISO 7783:2018 standard [41] was employed to calculate water vapor permeability (δ). The material diffusivity governed the length of the test, which took up to 8 days. The BS EN 1015-18 standard [42] was used to determine the water absorption coefficient which occurred due to the capillary action of the hardened mortars. The investigations were carried out under atmospheric pressure and specific conditions, using six cuboid samples per mortar type. When the constant mass of samples was achieved, epoxy resin was used to coat the four faces of each sample to reduce the influence of the external environment. In turn, the uncoated surfaces were immersed in water to the depth of 5–10 mm for 24 h (Figure 3 and Figure 4). Afterwards, the increase in mass was determined [43].

The OCA15 goniometer (Data Physics Instruments GmbH, Filderstadt, Germany) and a camera photographing the drops applied onto the sample surface, was used for measuring the water contact angle (CA) of liquid drops. This device has an automatic dosing system which enables the obtaining of precise and repetitive measurements. The measuring range is 0–180°/±0.1°. The water drops with the volume of 2 mm^3^ were applied using a micropipette [44]. Five drops were applied onto each sample, because of the mortar heterogeneity. The measurements were conducted twice: at the moment of drop application and after 5 min.

The frost resistance test was carried out following the saturation of samples with water after 28 days of maturation. Freezing was performed for 4 h at the temperature of −20 °C. Then, the samples were thawed for 4 h in water heated to +20 °C.

The mass loss was compared with the reference samples, which were stored in water at 20 ± 2 °C, in line with the PN-EN standard [45]. The considered samples underwent 50 freezing and thawing cycles (F–T).

In turn, the BS EN 12370:1999 standard [46] was employed to analyze the resistance to salt crystallization, by means of 40 mm × 40 mm × 40 mm samples. In the analysis, 14% sodium sulfate solution was used for dehydration. The results were obtained in the form of percentage corresponding to the difference between the obtained sample mass and its initial mass. Additionally, the number of cycles until destruction, i.e., complete lack of resistance to salt crystallization, was indicated. 

The microstructure, the interfacial transition zone (ITZ) between the cement paste and aggregate were investigated through a scanning electron microscope (SEM) analysis. The observation was carried out by means of FEG Quanta 250 microscope (FEI, Hilsboro, OR, USA). Photo scanning and X-ray analysis in the local mode and field mode were performed.

Statistical methods were applied for the analysis of the obtained results using STATISTICA 13.3 software (Statsoft, Tulsa, OK, USA). The dependences between the measured values—porosity and water absorption coefficient of cement mortars, absorptivity and compressive strength of cement mortars, frost resistance, compressive strength, and absorptivity—were established using the regression method. The coefficient of determination was also calculated for the regression line.

## 3. Results

### 3.1. Nanocellulose Characteristics

**Figure 5:**
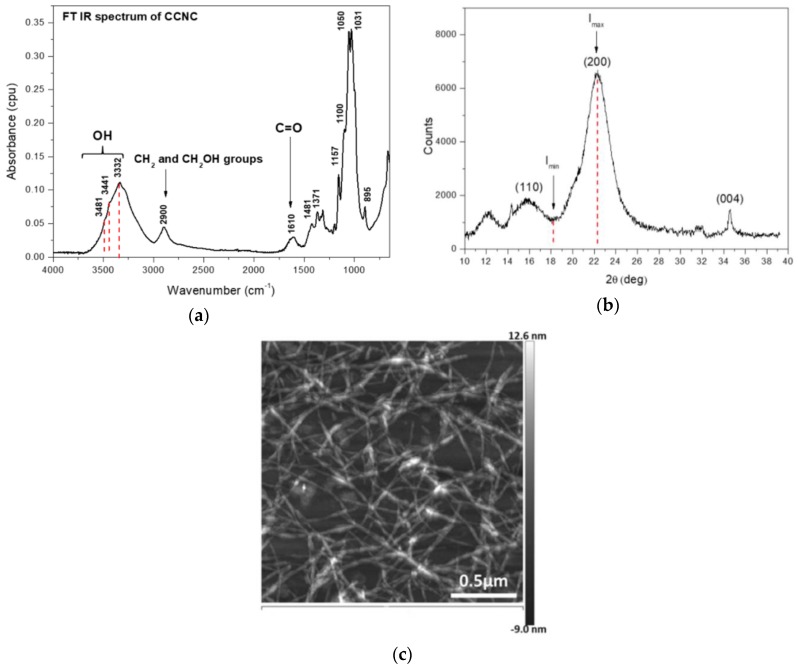
FT-IR spectrum of CCNC in the range of 4000–650 cm^−1^ (**a**); Diffractogram of CCNC (**b**); AFM image of CCNC (**c**).

CCNC was chosen because previously it was shown that the addition of nanocellulose with high crystallinity degree improves the hydrophobicity of concrete [31]. The nanocellulose molecular structure was characterized by means of FT-IR spectra (Figure 5a). The region of 3600–2750 cm^−1^ is characterized by OH and CH_2_ group vibrations: the band with maximum at 2900 cm^−1^ is related to vibration of CH_2_ and CH_2_OH groups, while broad band from 3600 to 3000 cm^−1^ is related to OH vibrations. The 1500–650 cm^−1^ region is particularly sensitive to stretching vibrations of C–O, C–C, ring structures, deformation vibrations of CH_2_OH groups. Apart from the presence of typical cellulose bands, the main characteristic feature is the presence of band at 1610 cm^−1^ related to the carbonyl groups. This indicates that the bleaching process with sodium hypochlorite leads to cellulose oxidation similar to those after TEMPO oxidation [47]. Cellulose and nanocellulose exhibit a tendency to form hydrogen bonds, which is enhanced by oxidation of surface [48]. This can result in better adhesion between nanocellulose and, for example, cement mortar particles.

The X-ray diffraction patterns of samples are presented in Figure 5b. There are clearly visible reflections at 2θ = ~15° (101), ~22° (200) characteristic for cellulose and 34.7° (004) characteristic for oxidized and ultrasonicated cellulose [47]. The calculated crystallinity degree of CCNC was 84%, which is comparable with the values obtained before for carrot pomace nanocellulose [33]. Moreover, the CCNC form large nanocrystals with length less than 1 μm and average diameter 3.31 nm [31]. Example AFM image obtained for CCNC is presented in Figure 5c.

It was also shown that the water CA of nanocellulose obtained from carrot is 64°, while the addition of chitosan caused an increase of the water CA to 98°, which was explained by increasing the interaction between chitosan and nanocellulose leading to lesser availability hydroxyl groups, and resulting in a lower hydrophilic nature of nanocellulose films [49].

### 3.2. Physical and Mechanical Properties of Cement Mortars

The properties of the cement mortars adopted for the investigation are shown in Table 3.

The addition of 1.5 nanocrystalline cellulose increased the bulk density of the material by 3.9% in relation to the reference mortar. Already the application of 0.5% biopolymer increased density by 1.7%. The addition of 1.5% nanopolymer improved the specific density of a mortar by 0.4%, whereas the 0.5% addition reduced the density by 1.1%, while in the case of 1% addition, the value remains unchanged. Nanocellulose decreases the porosity of cement mortars. This is because the polymer seals the mortar structure. The samples with 1.5% polymer were most sealed. Porosity decreased by 19.8% in relation to the reference mortar. This relationship was confirmed by the investigations of cement mortars with cellulose nanofibrils [50]. High specific surface area of nanomolecules increases the hydration degree and chemical reactivity [7]. Addition of nanomaterials to cements enables the desired distribution of molecules and improved material properties. In turn, an increase in the hydration degree reduces the porosity in cement paste. The cement sealing degree is strictly connected with water vapor permeability δ, which drops along with the increase in the nanopolymer content. Already a 0.5% (*v*/*v*) polymer addition reduces the water vapor permeability by 20.9%, while the maximum 1.5% dose decreases this parameter by 34.1%. In addition, the water absorption coefficient *C*_m_ drops as the amount of CCNC increases and is 32% lower for the CM1.5 mortars in relation to SM.

The composite strength can be increased by improving C–S–H gel creation in the cement matrix. This is demonstrated by the research conducted by Mazlan et al. [51]. Following CCNC addition, the cement composites become denser along with the C–S–H gel production, while small CCNC particles reduce the distance between fibrils. This results in good bonding between a polymer and cement matrix, thus improving the composite strength.

Studies indicated that an increase in compressive and flexural strength of mortars along with the amount CCNC. The flexural strength of CM1.5 mortar improved the most, by 10.9% in relation to the non-modified mortar. The flexural strength of CM0.5 and CM1 mortars increased by 3.4% and 8.1%, respectively. The highest compressive strength, i.e., 57.7 MPa was obtained by the CM1.5 mortar; this value is 27.6% greater than the strength of the SM mortar, which is equal to 45.2 MPa. The studies by Menezes-Silva et al. [52] on the nanocrystalline cellulose obtained from eucalyptus pulp as an addition to glass-ionomer cements indicated an increase in the compressive strength in all considered samples in the range of 24.5–33.1% and in the case of flexural strength—by 19.9%–52.2% in relation to the control samples. Cao et al. [18] observed an increase in flexural strength of the cement mortars modified with cellulose nanocrystals by 30% at 0.2% nanopolymer (*v*/*v*) in relation to the cement. The studies indicated that the hydration degree increased along with the CCNC content. Similar research was conducted by Hissaine et al. [53]. The results of studies on cement composite with cellulose nanofibrils showed that the modification with the polymer increases the hydration degree by 15%, which subsequently increased the flexural strength by up to 74%.

Ardanuy et al. [54] investigated the role of sisal fibers and cellulose nanofibrils on the mechanical properties of the cement composite. They reported that the cement composite with CNF was characterized by 40% higher flexural strength than the non-modified samples. Dai et al. [55] attributed the improved mechanical properties of cement composites with nanocellulose to the microcrack bridging mechanism due to the nanodimensions of the polymer and increased reciprocal effect of NFC molecules and cement compound caused by the reactivity of hydroxyl groups.

Shishehbor et al. [56] analyzed the influence of ITZ on the mechanical performance of the materials based on nanocrystalline cellulose. The authors observed that well-aligned CCNC lead to brittleness and catastrophic failure mechanism, while the naturally twisted interfaces improve the toughness of the material and facilitate obtaining the optimal mechanical performance.

The properties of cement composite with CCNC can be influenced by the source and method of CCNC production. Fu et al. [22] investigated the effect of adding nanocrystalline cellulose from different sources in CEMI/II and CEM V cements. The best results were obtained by adding CCNC in the amount of 0.2% CEM V by weight (a 49% improvement in flexural strength was obtained after 28 days, in relation to the reference samples). The authors believe that it has an effect of the nanomaterial production process. CCNC was acquired from acetate dissolving pulp in the course of oxidation with transient metal. Moreover, only CCNC exhibited a substantial improvement in flexural strength with CEMI/II cement, in relation to other samples.

**Figure 6:**
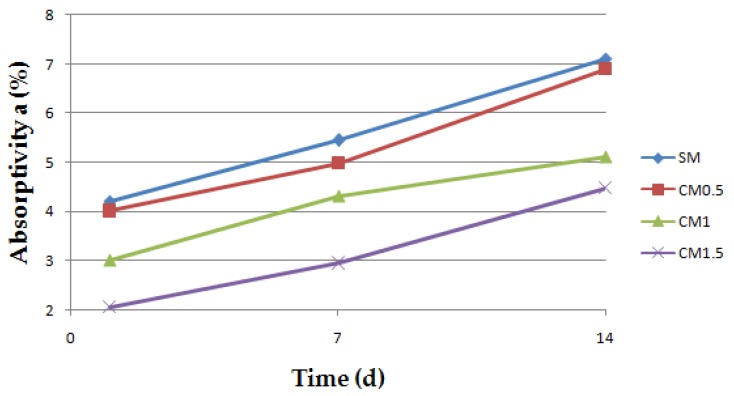
The changes in absorptivity of cement mortars in time.

To present the impact of CCNC on the changes in absorptivity in time, an experiment was conducted after 1, 7, and 14 days. The results are presented in Figure 6.

In the case of CM1.5, absorptivity is reduced by 32.4% after 14 days relative to the SM mortar. Studies indicated a drop-in absorptivity along with the content of cellulose nanocrystals. This results from the sealing of the paste-aggregate ITZ by the nanopolymer, which is confirmed by the studies conducted by Aguiar et al. [57]. The greatest drop in absorptivity was observed in the case of CM1.5; after the first day of the research, it was 51.3% lower in relation to the reference sample. This difference decreased in time. Following 7 days, the absorptivity of the CM1.5 sample in relation to the non-modified sample is 45.8% lower, whereas after 14 days, the difference amounts to 36.9%. This proves the greatest effect of the admixture in the first days of the investigations. The results are confirmed by the previous studies of Barnat-Hunek et al. [31], in which the mixtures containing nanocrystalline cellulose exhibited superior properties lowering the absorptivity of the concrete. The absorptivity of the samples with 1% CCNC (*v*/*v*) dropped by 64.3% in relation to the reference samples.

The CA was measured in a goniometer. Table 4 shows the CA at the moment of water drop application and after 5 min.

CA increases along with the admixture content. The highest angle, equal to 114°, was obtained for the CM1.5 mortar. It is 5-fold greater than for the non-modified mortar. The CA decreases with time. The most significant drop was observed for the SM mortar, in which the CA dropped by 60% already after 5 min. In the case of the mortars containing admixtures, the CA decreases much slower. A clear relationship between the CA decrease rate and the amount of admixture in mortars should be noted. After 5 min, the CA in the CM0.5 mortar it reduced by 38.2%, whereas in CM1.5 only by 8.7%. Despite the passing time, the beneficial effect of nanocellulose addition on the considered property could still be observed. This was confirmed by the research by Barnat-Hunek et al. [31]. The mortars with cellulose nanocrystals CCNC admixture exhibited an increase in the CA which occurred along with added polymer content. The 1% biopolymer addition raised the CA by 73.8% in relation to the reference mortars. This team also investigated the mortars with cellulose nanofibrils. Similarly, an increase in the CA was noted; however, this result was slightly lower than in the case of nanocrystal application. In this case, the CA increased by 67.8%.

**Figure 7:**
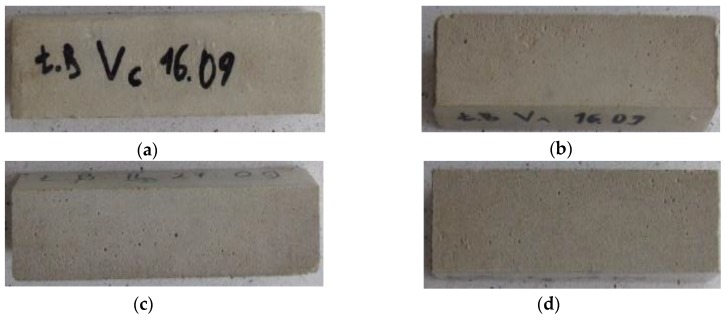
Condition of representative samples before the frost resistance test: (**a**) SM; (**b**) CM0.5; (**c**) CM1; (**d**) CM1.5.

**Figure 8:**
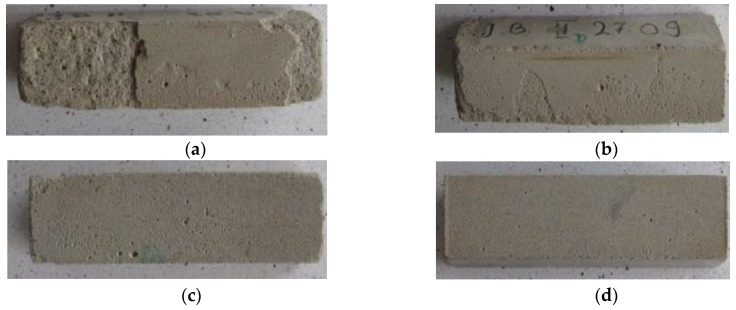
Condition of representative samples after the frost resistance test along with the mean mass loss: (**a**) SM; (**b**) CM0.5; (**c**) CM1; (**d**) CM1.5.

Figure 7 and Figure 8 show the visual condition of samples before and after the frost resistance test (50 freezing and thawing cycles F–T) as well as the mass loss.

A clear correlation between the percent mass loss and the CCNC content can be seen following the frost resistance test. Along with the increase of the admixture content, the mass loss was significantly reduced. The lowest mass loss following 50 freezing and thawing cycles, i.e., 0.1%, was exhibited by the CM1.5 samples, which is 98% better result in comparison with the SM mortars. Already a 0.5% admixture addition resulted in a five-fold reduction in mass loss, whereas increasing the dose to 1% decreased the loss by the factor of 25. An improvement in frost resistance resulted from the sealing of composite microstructure by nanomolecules. The studies conducted by Behfarnia and Salemi [58] on the concrete with the addition of nanosilica and aluminum oxide nanoparticles confirmed this theory. According to the authors, the nanopolymer fills the voids between the C–S–H gel particles. The beneficial influence of nanopolymers on the frost resistance of concrete is also reported in the studies on graphite nanoparticles [59]. The concrete containing graphite nanoadditives in the amount of 0.01% by weight can resist a greater number of F–T cycles and higher temperatures. An improvement in frost resistance was also noted in the case of cement composites with nanocellulose [31]. Following 100 F–T cycles, a clear decrease in mass loss occurred in the concretes with nanocrystalline and NFC. Moreover, the mass loss decreased along with the increase in the amount of added nanopolymer.

**Figure 9:**
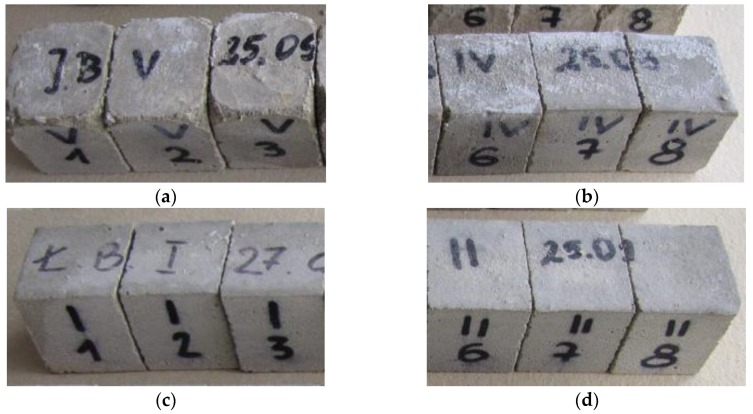
Mass loss after salt crystallization test (%): (**a**) SM—3.5%; (**b**) CM0.5—1%; (**c**) CM1—0.1%; (**d**) CM1.5—0.1%.

Figure 9 presents the visual condition of the samples following the investigation as well as the mass loss after the salt crystallization test.

After the salt crystallization test, the lowest mass loss, equal to 0.1%, was noted in the CM1.5 mortars. As in the case of the frost resistance test, the best results (0.1%) were observed for the CM1 and CM1.5 mortars. A 1.5% admixture addition reduced the mass loss resulting from the negative impact of aggressive salts by the factor of 35. Already the lowest admixture amount, equal to 0.5%, reduced the mass loss by 71.4% in relation to the reference samples. Goncalves et al. [50] subjected mortars to a sulfate trial. The results proved that nanocellulose hinders the infiltration of sodium sulfate ions to cement mortars in a clear way. According to the authors, this feature contributes to a reduction in ettringite formation, while the cement with cellulose nanofibrils admixture exhibits a comparable resistance to sulfates to the sulfate resistant cements. An improvement in the compressive strength and pore tightening in the mortar was observed as well.

**Figure 10:**
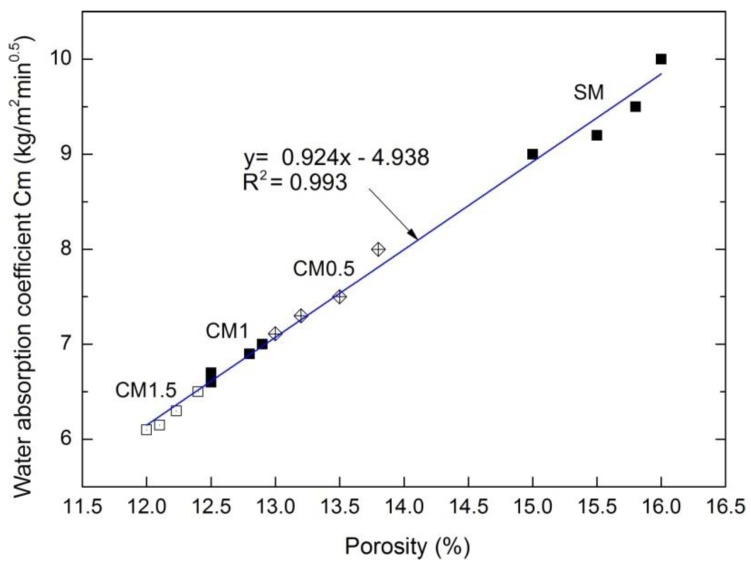
Correlation between porosity and water absorption coefficient Cm of cement mortars.

**Figure 11:**
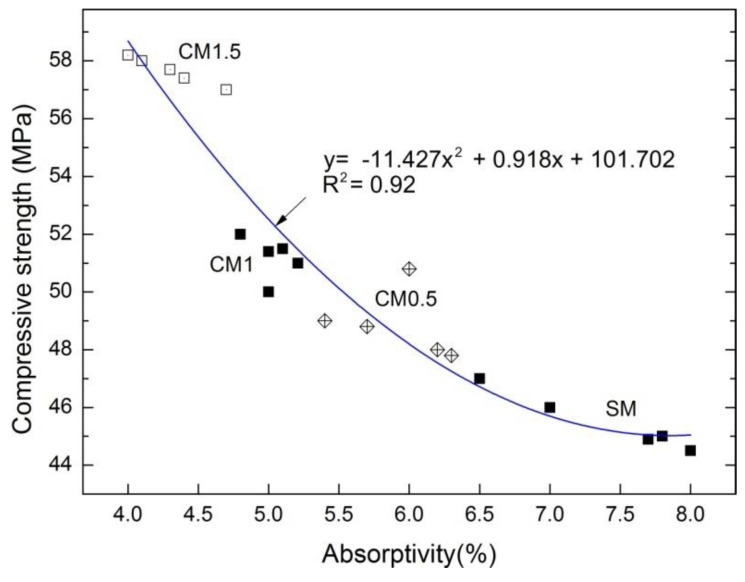
Correlation between absorptivity and compressive strength of cement mortars.

The obtained results indicated strong correlations between certain mortar properties depending on the nanocellulose content (Figure 10 and Figure 11). To demonstrate the relationship between the water absorption coefficient C_m_ and porosity, a simple linear regression model was employed (Figure 10).

Figure 10 presents a strict linear dependency between the porosity and water absorption coefficient Cm. These features are directly dependent on each other, as evident from a very good determination coefficient *R*^2^ = 0.993. The higher the porosity, the greater the water absorption coefficient.

Compressive strength is strictly dependent on the absorptivity of cement mortars with/without nanocellulose and on the cellulose content, which was presented in Figure 11. The higher the cellulose content, the greater the compressive strength of mortars. This relation is expressed in the following polynomial function: $y=-11.427x^{2}+0.918x+101.702$. A good determination coefficient *R*^2^ = 0.92 was obtained.

**Figure 12:**
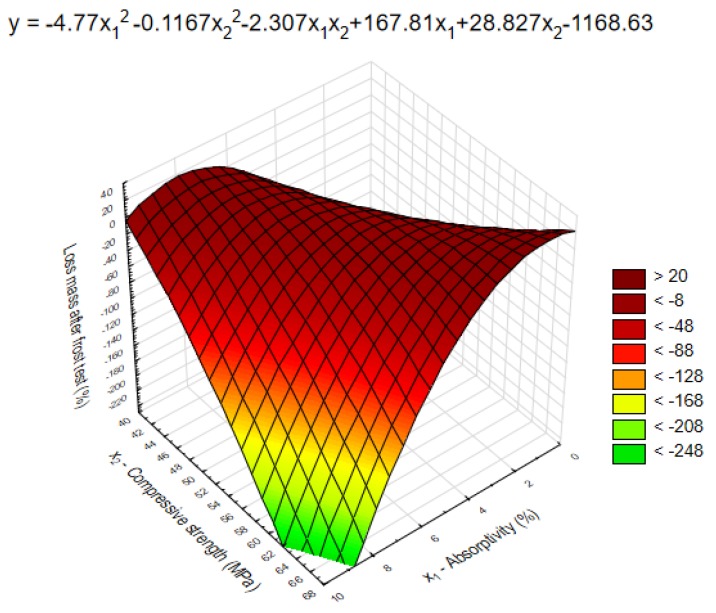
Dependence of frost resistance on compressive strength and absorptivity.

The model presented below (Figure 12) shows the range in which the properties of mortars with nanocellulose influence the frost resistance, which indirectly determines the corrosion resistance of a material. Figure 12 presents the model, as well as the frost resistance of mortars depending on two variables: x_1_—compressive strength and x_2_—absorptivity.

Based on Figure 12, Table 3 and Figure 8, it can be concluded that frost resistance is closely related to the compressive strength and absorptivity of cement mortars with nanocellulose. The lower the strength, the lower the frost resistance and high absorptivity. Knowing these relationships can be useful not only in practice when choosing the right amount of nanocellulose admixture, but can also be used as a basis for designing the composition of cement mortars for the walls exposed to frost.

**Figure 13:**
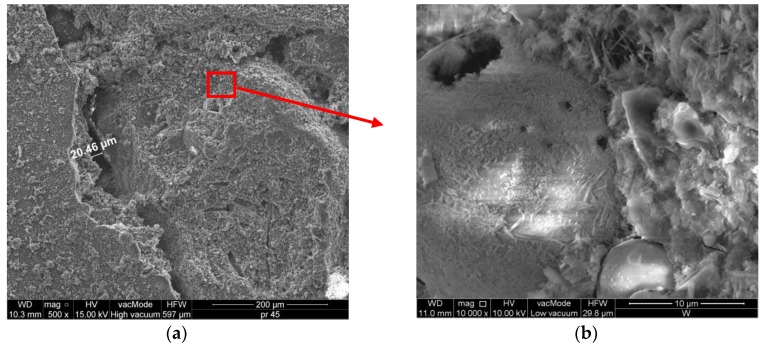
Microstructure of the SM mortars: (**a**) SM, 500×; (**b**) SM, 10,000×.

**Figure 14:**
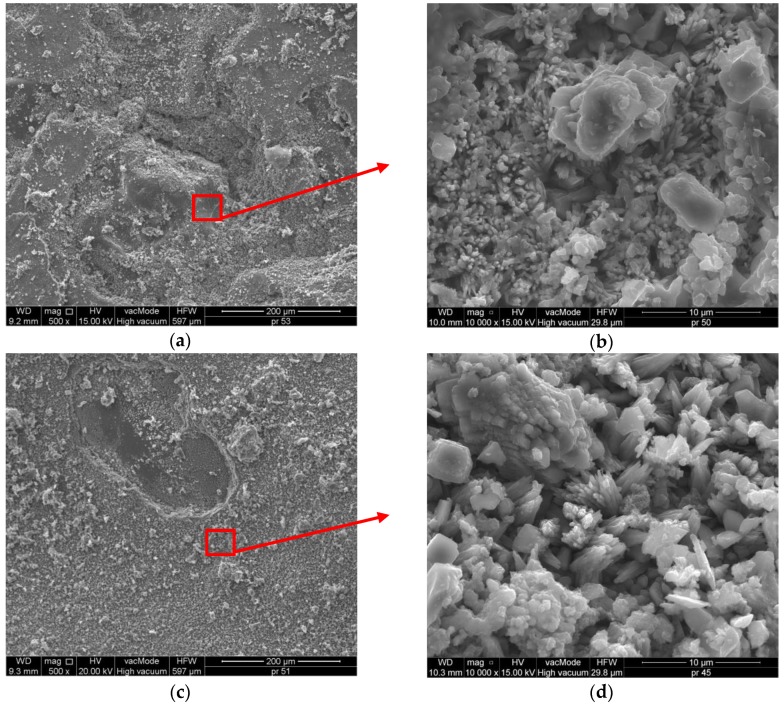
Microstructure of CM with nanocellulose: (**a**) CM0.5, 500×; (**b**) CM0.5, 10,000×; (**c**) CM1.5, 500×; (**d**) CM1.5, 10,000×.

In the microstructural analyses of mortars (Figure 13 and Figure 14), the samples collected from the undamaged cuboid parts from the bending test, unaffected by the load, were investigated. Cracks in mortars are relatively common phenomenon since they are a natural property of mortars and their structure. The ITZ of a cement matrix is usually regarded as the weakest element of a concrete or mortar. The material cracking and its destruction begins there, as described by Golewski [60]. It is believed that cracks, regardless of their properties, may constitute the point of entry for water or aggressive chemical ions, which infiltrate into the concrete structure, deteriorating its condition and durability. This means that a material with larger cracks is characterized by greater permeability and thus lower durability, as indicated by Kockal et al. [61].

The SM structure (see Figure 13a) is not compacted enough; cracks with the width up to 20.46 µm occur in the cement paste while sand grains are weakly bonded with the mortar (ITZ). This relates to the highest absorptivity among the considered mortars and the lowest frost resistance (mass loss of 5.1%) (See Figure 8). Figure 10b showing the mortar structure in 10^4^ magnification presents the hydration products in the SM mortar, which are mainly comprised of irregular, flat particles forming tight clusters corresponding to the C–S–H phase. Hardened cement paste prepared from the Portland cement mainly consists of C–S–H phases (approximately 70%) and calcium hydroxide as well as aluminate and calcium aluminoferrite hydration products. Based on the literature [62], C–S–H gel can exhibit three morphologies: fibrous-acicular form (type I), reticule or honeycomb form (type II) and denser-almost spheres form (type III). In the cement hydration period, the pores of hardened cement paste are filled with short fibers or phases of hydrated calcium silicates. The characteristic property of this phase is the transformation of calcium aluminate trisulfate 3CaO·Al_2_O_3_·3CaSO_4_·32H_2_O to calcium aluminate monosulfate 3CaO·Al_2_O_3_·CaSO_4_·12H_2_O [63]. The ettringite crystals usually assume elongated, needle-like forms (Figure 13b).

Figure 14 presents the microstructure of the CM mortar with nanocellulose. The structure and ITZ of the CM mortars are different from SM. The mortar is tight and shows no cracks or scratches (see Figure 14a,c). The structure of the SM mortar shows pores and cracks, while SM is very tight and no pores can be seen, which is reflected in the investigations of physical properties (Table 3). In a 10^4^ magnification, a completely different structure from the SM mortars can be seen (Figure 14b,d).

The structure and ITZ of the CM mortars are different from SM. The mortar is tight and shows no cracks or scratches (see Figure 14a,c). The structure of the SM mortar shows pores and cracks, while SM is very tight and no pores can be seen, which is reflected in the investigations of physical properties (Table 3). In a 10^4^ magnification, a completely different structure from the SM mortars can be seen (Figure 14b,d).

In the mortars with nanocellulose, the morphology of C–S–H phase samples indicates both irregular, flat particles, as well as a well-crystallized C–S–H structure (Figure 14b,d). In turn, Figure 10b,d show a massive, lamellar structure of hexagonal crystals—portlandite Ca(OH)_2_. A well-developed portlandite lamella and C–S–H structure can be observed both in SM as well as in CM (Figure 13b, Figure 14b,d). Thus, it can be stated that the combination of cement mortars with nanocellulose influences its durability, as indicated in other studies [31]. No ettringite can be observed in the CNC mortar, which is particularly evident in the SM reference mortar. In the CM concrete, a very good bonding of the cement paste with sand was responsible for the superior concrete durability. The CM1.5 samples are characterized by a lower number of pores and microcracks in the structure than the SM mortar, which is also confirmed by the investigations pertaining to the physical properties (Table 3). This mortar is characterized by the lowest absorptivity, open porosity, and mass loss following 50 F–T cycles. Similar observations were reported by Barnat-Hunek et al. [31].

The chemical composition of the analyzed mortars was presented in Table 5.

The analysis of chemical composition indicates that silica, as well as calcium and aluminum oxides, are found in all mortar types. Out of the analyzed mortars, the highest SiO_2_ content was observed in CM1.5. The content of Fe_2_O_3_ also increases in the mortars with CNC admixture.

## 4. Conclusions

The physical properties and microstructure of cement mortars with CCNC were investigated. The following conclusions can be drawn based on the results and discussions presented in this paper:

- Addition of nanocrystalline cellulose to a mortar contributes to an increase in bulk and specific density. A 1.5% admixture increases bulk density by 3.9% and specific density by 0.4%, in relation to the reference mortars.
- CCNC seals cement mortars. In the case of the mortars with 1.5% admixture, the porosity reduced by 19.8% in comparison with the reference mortars.
- An increase in the nanopolymer content reduces the water vapor permeability by 34.1% in the CM1.5 mortars.
- Water absorption coefficient C_m_ is reduced as the amount of CCNC increases and is 32% lower for the CM1.5 mortars in relation to SM.
- In the case of the CM1.5 mortar, the nanocrystalline cellulose addition improves the compressive and flexural strength by 27.6% and 10.9%, respectively.
- Along with the increase in the biopolymer content, the absorptivity of cement mortars is reduced. The greatest reduction was observed in the case of the CM1.5 mortar, in which a 51.3% reduction in absorptivity—in relation to SM—was noted after the first day of the investigation.
- CA increases along with the CCNC admixture amount. The highest angle, equal to 114° was obtained for the CM1.5 mortar. A lower rate of CA reduction was also observed in the mortars with admixtures.
- As the amount of admixture is increased, the mass loss following the frost resistance and sulfate crystallization tests reduces. After 50 F–T cycles of the CM1.5 mortar, a 98% improvement was noted in relation to the reference samples. In turn, the crystallization tests indicated a 35-fold decrease in mass loss following a 1.5% nanopolymer addition.
- The SEM investigations showed that the CM1.5 samples are characterized by a much lower number of pores and microcracks in the structure the SM mortar. In the mortars with CCNC admixture, the formation of ettringite was not observed, in contrast to the SM reference mortar.

Cellulose and its nanoderivatives are employed in numerous branches of the economy. Unfortunately, its application in the construction industry is still inadequate. Nanocellulose, being a biodegradable and eco-friendly material, should be used at a large scale.

In the future, the authors plan to conduct further studies on lightweight mortars with NFC from apples and microcrystalline cellulose from wood pulp. The authors believe that lightweight mortars with the addition of these nanopolymers obtain better strength and hydrophobizing properties than standard cement mortars with their addition.

## Figures and Tables

**Table 1:** Compositions of cement mortars (kg/m^3^).

Component (kg/m^3^)	SM0	CM0.5	CM1	CM1.5
Portland cement CEM I 32.5 R	265	265	265	265
Sand 0–2.0 mm	1405	1405	1405	1405
Nanocellulose additive	-	1.325	2.65	3.975
Nanocellulose additive (%)	-	0.5	1	1.5

**Table 2:** The CEM I 32.5R Portland cement parameters [31].

Parameters	Unit	Value
Specific surface	(m^2^/g)	0.3985
Density	(g/cm^3^)	3.05
Compressive strength	(MPa)	-
after 2 days	(MPa)	17.6
after 28 days	(MPa)	43.2
Volume stability	(mm)	<10
Loss on ignition by cement weight	(%)	5.0

**Table 3:** Properties of the cement mortars.

Properties	Type of Mortars			
	SM	CM0.5	CM1	CM1.5
Bulk density ρ_a_, (g/cm^3^)	2.22	2.27	2.28	2.31
Specific density ρ (g/cm^3^)	2.63	2.61	2.63	2.64
Total porosity P (%)	15.57	13.32	12.83	12.49
Water vapor permeability δ (10^−12^ kg/m·s·Pa)	9.1	7.2	6.8	6.0
Water absorption C_m_ (kg/m^2^min^0.5^)	0.34	0.32	0.29	0.23
Compressive strength (MPa)	45.2	48.1	51.1	57.7
Flexural tensile strength (MPa)	5.7	5.9	6.2	6.4

**Table 4:** Contact angle of concretes at the moment of water drop application and after 5 min.

Type of Mortars	Contact Angle CA (°)	
	t_1_ = 0 s	t_2_ = 5 min
SM	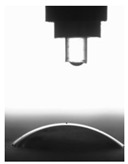 CA = 30°	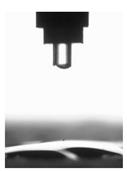 CA = 12°
CM0.5	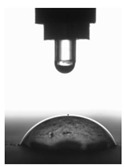 CA = 47°	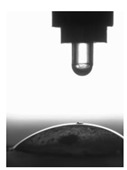 CA = 29°
CM1	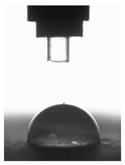 CA = 89°	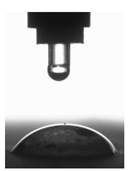 CA = 52°
CM1.5	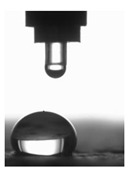 CA = 114°	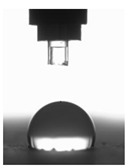 CA = 104°

**Table 5:** Chemical composition of the analyzed mortars.

Samples		Compound								
		Al_2_O_5_	SiO_2_	Na_2_O	Fe_2_O_3_	MgO	K_2_O	CaO	P_2_O_5_	SO_3_
SM	Content [wt %]	39.75	48.06	0.65	3.99	1.85	2.41	3.29	-	-
CM0.5		25.21	57.21	0.89	10.76	1.75	2.21	1.03	-	0.34
CM1		20.06	58.69	1.18	14.79	1.60	2.53	1.33	-	-
CM1.5		22.75	59.05	1.40	10.62	1.21	3.54	1.31	-	0.62
